# Examining attachment, cortisol secretion, and cognitive neurodevelopment in preschoolers and its predictive value for telomere length at age seven

**DOI:** 10.3389/fnbeh.2022.954977

**Published:** 2022-10-13

**Authors:** Euclides José de Mendonça Filho, Ariane Frechette, Irina Pokhvisneva, Danusa Mar Arcego, Barbara Barth, Camila-Andrea Valle Tejada, Roberto Sassi, Ashley Wazana, Leslie Atkinson, Michael J. Meaney, Patricia P. Silveira

**Affiliations:** ^1^Department of Psychiatry, McGill University, Montreal, QC, Canada; ^2^Ludmer Centre for Neuroinformatics and Mental Health, Douglas Hospital Research Center, Verdun, QC, Canada; ^3^Integrated Program in Neuroscience, McGill University, Montreal, QC, Canada; ^4^Division of Child and Adolescent Psychiatry, University of British Columbia, Vancouver, BC, Canada; ^5^Department of Psychology, Ryerson University, Toronto, ON, Canada; ^6^Department of Psychology, Toronto Metropolitan University, Toronto, ON, Canada; ^7^Singapore Institute for Clinical Sciences, Agency for Science, Technology and Research (A*STAR), Singapore, Singapore

**Keywords:** attachment, cortisol, neurodevelopment, telomere length, preschooler

## Abstract

**Background:**

Secure attachment reflects caregiver-child relationship in which the caregiver is responsive when support and comforting are needed by the child. This pattern of bond has an important buffering role in the response to stress by the reduction of the negative experience and its associated physiological response. Disruption of the physiological stress system is thought to be a central mechanism by which early care impacts children. Early life stress causes cellular and molecular changes in brain regions associated with cognitive functions that are fundamental for early learning.

**Methods:**

The association between attachment, cortisol response before and after the Strange Situation Experiment, and neurodevelopment was examined in a sample of 107 preschoolers at age three. Also, the predictive effect of cortisol reactivity and attachment on telomere length at age seven was investigated in a followed-up sample of 77 children.

**Results:**

Children with insecure attachment had higher cortisol secretion and poorer neurodevelopmental skills at age three. A significant cortisol change was observed across the experiment with non-significant interaction with attachment. The attachment and neurodevelopment association was not mediated by cortisol secretion. Preschoolers’ attachment and cortisol did not associate nor interacted to predict telomere length at age seven.

**Conclusion:**

These findings add evidence to the detrimental effects of insecure attachment as an aggravator of the physiological response to stress and poorer neurodevelopment during the preschool period. Although attachment and cortisol were not predictive of telomere length, intervention policies that promote secure attachment are more likely to positively echo on several health domains.

## Introduction

Positive parent-child relationship is crucial for optimal child development. Attachment behavior, conceived as a behavioral system, is thought to have an evolutionary adaptive value and biological function (Ainsworth et al., 2015; Cassidy, 2016). Individual variations of attachment styles define the degree to which there are emotional availability, consistent attention, and accurate interpretation of the child’s signals in the child-caregiver relationship (Finzi et al., 2001; Alvarenga et al., 2019). This in turn will influence how the child will develop certain behavioral, psychological, and emotional concepts to understand and react to social interactions later in life (Bowlby, 1988; Finzi et al., 2001). Along these lines, a secure attachment relationship between the child and the caregiver consists of one of the most important forms of stress buffer driven by the reduction of negative and maintenance of positive emotions, and the physiological processes associated with the emotional experiences (Roque et al., 2012; Reilly and Gunnar, 2019).

Disruption of the physiological stress response system is thought to be a central mechanism by which early care and parenting negatively impact child development (Ashman et al., 2002; Meaney and Szyf, 2005; McLaughlin et al., 2015; Reilly and Gunnar, 2019). The hypothalamic-pituitary-adrenal (HPA) axis plays a central role in controlling and regulating the relationship between the central nervous system and the peripheral stress response (Kudielka and Kirschbaum, 2005; Badanes et al., 2012; Reilly and Gunnar, 2019). Activation of the HPA axis in response to stress results in the release of cortisol to mobilize energy and resources to respond to environmental threats (Kudielka et al., 2004). This mobilization allows the organism to maintain functioning under challenging conditions (allostasis), however, under conditions of repeated and/or chronic stress (high allostatic load), typical HPA function might be disrupted in the long term (Reilly and Gunnar, 2019). Sensitive and responsive caregiving is strongly linked to the development of secure attachment (Gervai, 2009) which acts as a buffer to stressful exposures (Badanes et al., 2012; Fong et al., 2017; Johnson et al., 2018). Deprivation of sensitive and responsive caregiving in early childhood could be especially harmful when exposure to an adverse early environment and neglect is also present (Shakiba and Raby, 2021). Evidence indicates that caregiver’s presence reduces activation of the HPA axis in offspring exposed to a stressor, and children raised in a predominately negligent environment exhibit altered autonomic nervous system function (Zeanah et al., 2005; McLaughlin et al., 2015; Nelson and Gabard-Durnam, 2020). In addition, animal models demonstrated that stress during early development causes various cellular and molecular changes in the brain, mostly seen in the hippocampus and frontal regions (Meaney and Szyf, 2005; McEwen and Gianaros, 2010). Those regions are associated with complex cognitive functions that are fundamental for learning, such as intelligence, executive functions, and memory (Houdé et al., 2010; Ziegler et al., 2013; Modabbernia et al., 2020). However, a great deal of work on attachment theory focused on infants’ emotional regulation (Schore, 2001; Roque et al., 2012), anxiety and fearfulness (Bergman et al., 2008; Colonnesi et al., 2011), and externalizing behavior problems (Finzi et al., 2001; Nofech-Mozes et al., 2019).

There is evidence that secure attachment favors a better performance on executive functioning tasks in infants (Menon et al., 2020), and meta-analytic data indicating a strong association with language and a weaker but significant association with intelligence (IJzendoorn et al., 1995). However, investigations about how attachment styles influence preschoolers’ HPA axis response and emergent cognitive abilities, which are the foundation of early learning, are still scarce. Preschool education benefits the development of early skills that predict short and long-term effects on school readiness and health outcomes (Yoshikawa et al., 2013). Attachment in infancy has been particularly explored in the context of developmental pathways, in which attachment classification posits as a relevant indicator of future psychopathology by marked continuity (Crittenden, 1992; Moss et al., 2005). However, although attachment in infancy predicts attachment patterns in preschool years, overall attachment stability classification is considered to be moderate (Moss et al., 2005; Opie et al., 2020). Therefore, the large literature on attachment, cortisol reactivity, and child outcomes in early infancy may not be directly generalized to the preschool developmental stage. This indicates a need to consider attachment patterns in preschool-age with special attention to its specificities, such as children’s considerably more complex cognitive and linguistic skills, and the challenges associated with dealing with a new environment and separation from the main caregiver (Crittenden, 1992; Moss et al., 2004).

Insecure attachment in the preschool period was associated with poorer cognitive skills (O’Connor and McCartney, 2007) and increased cortisol across the childcare day (Badanes et al., 2012). However, studies exploring the moderation or mediation role of HPA functioning in preschoolers are still lacking. This is of particular relevance as cortisol reactivity is considered an important biomarker of individual differences, moderating the association between attachment and cognitive outcomes, and parental support in the relative change in anxious attachment styles (Van Bakel and Riksen-Walraven, 2004; Fong et al., 2017; Nofech-Mozes et al., 2019; Houbrechts et al., 2021). More importantly, as a precursor of early social development, attachment patterns are likely to obtain promising effects on several health domains through a cascade of positive adaptation by caregiver-child dyads (Dozier et al., 2008; Newman et al., 2015; Handley et al., 2017; Julian et al., 2018).

Another biomarker sensitive to stressful life experiences that increase HPA axis activation, oxidative stress, and inflammation is telomere length (TL; Houben et al., 2008; Gotlib et al., 2015; Martens et al., 2020). TL is composed of sequences of repetitive DNA that protect chromosomal ends fusion and degradation (Houben et al., 2008). The impossibility of DNA polymerases to replicate chromosomes completely leads to the shortening of chromosomes at each cell division and, therefore, is considered an indicator of cellular aging (Houben et al., 2008; Martens et al., 2020). Cross-sectional studies have found that parental socioeconomic status and depression levels are associated with accelerated TL reduction in newborns (Martens et al., 2020) and pre-adolescents (Gotlib et al., 2015). While shorter TL was associated with higher cortisol reactivity to stressful settings (Gotlib et al., 2015), secure attachment in early life seems to act as a moderator of the effects of cumulative childhood adverse experiences on adults’ TL (Dagan et al., 2018). Even so, there is a paucity of studies investigating the predictive feature of early attachment styles and HPA axis response on TL at a later developmental stage.

For the reason of the lack of studies concerning the long-term effects of attachment styles and cortisol response during preschool years, the current study aimed to investigate if the associations of attachment styles with HPA axis functioning and cognitive neurodevelopment are also observed in preschool years, as well as the predictive effect of cortisol reactivity and attachment on telomere length at age seven. For this, we investigated the impact that different attachment styles have on cortisol responses across the Strange Situation Experiment (Moss et al., 2004), and if differences between secure and insecure attachment styles predict neurodevelopment of these infants assessed by the Bayley Scales of Infant Development (BSID; Bayley, 1993). Because individual variations in cortisol secretion are thought to be a mediator between early care environment and developmental outcomes (Reilly and Gunnar, 2019), we analyzed a mediation model with aggregated cortisol as a mediator between attachment style and neurodevelopment. We also investigated if attachment style and HPA axis response at 3 years of age interacted to predict children’s TL at 7 years of age. Other co-factors were also considered, including maternal education and levels of depression, and family income as possible confounding of the association of attachment, cortisol response, neurodevelopment, and telomere length.

## Materials and methods

### Participants and procedures

A sample of 107 mother-child dyads was drawn from the Maternal Adversity, Vulnerability, and Neurodevelopment study (MAVAN; O’Donnell et al., 2014). MAVAN is a Canadian community-based longitudinal and prospective cohort following mothers and their infants. The MAVAN project was established in 2003 with an initial sample of 630 mother-child dyads that were monitored to investigate the potential consequences of the prenatal (in combination with the postnatal environment) in children’s neurodevelopmental outcomes. Children were monitored from birth to 72 months of age using several measures of neurodevelopment. Pregnant women were recruited around 13 to 20 weeks of gestation from obstetric clinics in hospitals. They were eligible to take part in the study if they were over 18 years of age, fluent in either English or French and did not have: (1) serious obstetric complications during the pregnancy or delivery of the child; (2) child birth weight <2000 g; (3) prematurity (<37 weeks’ gestation) or; (4) any congenital diseases. Data and sample size from this study are based on sample availability at the 3-year post-partum visit (*N* = 107) the follow-up subsample (*N* = 71) from the 7-year visit. Data were collected at the St-Joseph’s Hospital (Hamilton-ON, Canada). The average maternal age at the 3-year visit was 33.8 years (SD = 4.8), preschoolers’ age was 3.7 years (SD = 0.1) and 60.7% of participants were boys. At the 7-year visit, the average maternal age at data collection was 35.9 years (SD = 5.1) and children’s age was 7.27 (SD = 1.4, 52.1% boys). Ethical approval for this study was obtained from the St-Joseph’s Hospital and McGill University, and informed written consent was obtained from each mother.

### Measures

#### Strange situation experiment and infant attachment style

A modified version of the Ainsworth’s preschool strange situation experiment was used to define the attachment style of infants at the age of 36 months (Moss et al., 2004). The experiment consisted of a 20-min observational assessment of four stages with varying duration of 3–5 min each. At the first stage, the mother and child were separated, and the child was left alone in the room with age-appropriate toys. In the second stage, they were reunited and freely interact with each other. In the third stage, the dyad was separated again, and in the last stage, they met again. The mothers received no specific instructions concerning the reunions.

Trained coders classified the reunion styles based on evaluations of the child’s physical proximity to the mother, affective expression, and verbal interactions. Securely attached children use the mother to explore the playing room, thus children were classified with secure attachment (*N* = 61) if a pattern of relaxed and mutually enjoyable interactions between the dyad was observed. Children were classified as insecure-avoidant (*N* = 9) if a pattern of physical and affective avoidance toward the parent was exhibited. Insecure-avoidant children will typically not be engaged in the caregiver’s verbal initiatives. Parent-child verbal exchanges are often short, with little elaboration followed by topics initiated by the other. Children classified as insecure-ambivalent/dependent (*N* = 16) struggled to initiate independent behaviors – followed the caregiver around the room or wanted to be held often – or exhibited excessive immaturity evidenced by passivity or conflictual behavior patterns. Interactions between parent and child often seem to interfere with child exploration. Children classified as insecure-disorganized (*N* = 21) were unable to use the caregiver as a secure base for exploration but did not clearly show the insecure-avoidant or insecure-ambivalent/dependent pattern. We opted to combine the insecure attachment sub-types (avoidant/dependent, ambivalent, and disorganized) into a general insecure category (*N* = 46) due to the small number of subjects in each insecure group.

#### Salivary cortisol

Infant saliva was sampled for cortisol determination at four points during the 36-month cohort visit. The first saliva sample was collected 20 min before the Strange Situation Experiment. The second, third, and fourth saliva samples were collected 20-min, 40-min, and 60-min, respectively, after the end of the experiment. Saliva sampling was performed using Visispear sponges (Beaver-Visitec Int., Waltham, MA, USA) and samples were stored in centrifuge tubes at −80°C until assayed. Cortisol assays were performed in the Neurosciences Laboratory at St. Joseph’s Healthcare, Hamilton, ON, under the guidance of the laboratory manager. Samples were thawed, and saliva was extracted *via* centrifugation (3,000 rpm at room temperature for 15 min) yielding approximately 200 μl of saliva. Salivary cortisol measurements were quantified using a high-sensitivity enzyme immunoassay kit (Salimetrics, LLC, State College, PA, USA). According to Salimetrics’ assay protocol^1^ “the correlation between saliva and serum was highly significant, *r* (47) = 0.91, *p* < 0.0001” (p. 16), indicating that “salivary cortisol levels reliably estimate serum cortisol levels” (p. 3). Assay sensitivity was 0.08 nmol/L, and the intra- and inter-assay coefficients of variation were 3.5 and 5.1%, respectively.

#### Child neurodevelopment

The Mental Scale of Bayley Scales of Infant and Toddler Development: Second Edition (BSID; Bayley, 1993) was used as a measure of neurodevelopment. The Bayley Scales mental development index (MDI) is a composite of children’s language and cognitive abilities. It assesses age-appropriate levels of memory, problem-solving, habituation, incipient number concepts, generalization, classification, vocalizations, and language skills, through a series of age-ordered tasks. Raw scores are converted to a standardized measure of cognitive development considering each age group with a mean of 100 and a standard deviation (SD) of 15. The psychometric properties of the Bayley scales indicated good to excellent validity and reliability (Da Silva et al., 2018). Children’s development assessment was performed by trained and experienced professionals.

#### Telomere length

Catch-All*™* Sample Collection Swabs (Epicentre^®^, Madison, WI, USA) were used to collect buccal cells. Genomic DNA was extracted starting with a lysis step as indicated by the collection kit manufacturer’s instructions, then using QIAsymphony DNA kits (DSP DNA midi kit; Qiagen, Hilden, Germany), with an additional digestion step of Qiagen RNase-A. DNA concentration and quality were assessed using the MBI P330 nanophotometer (Implen, Munich, Germany). Extracted genomic DNA was diluted, for a final concentration of 15 ng per sample. The telomere PCR reaction mixture, final volume 15 μl, contained: 5 μl of 2× LightCycler^®^ 480 SYBR Green I Master (F. Hoffmann-La Roche AG, Basel, Switzerland) for a final concentration of 1×; 1.2 μl of primers TelgF (ACACTAAGGTTTGGGTTTGGGTTTGGGTTTGGGTTAGTGT) and TelcR (TGTTAGGTATCCCTATCCCTATCCCTATCCCTA-TCCCTAACA; Cawthon, 2009), 0.6 μl of each, at final concentration of 1 μM each; 2.3 μl of water; and 1.5 μl of sample for a final DNA amount of 22.5 ng. The single-copy genome PCR reaction contained the exact amount and concentration of reagents except for the primers 36B4U (CAGCAAGTGGGAAGGTGTAATCC) and 36B4 (DCCCATTCTATCATCAACGGGTACAA) (O’Callaghan and Fenech, 2011).

To generate absolute values, standard curves for telomere and single-copy genome were performed. The oligomer standard, 84 bp in length (TTAGGG) repeated 14 times, and the synthesized 36B4 oligomer standard, 75 bp in length (CAGCAAGTGGGAAGGTGTAATCCGTCTC-CACAGACAAGGCCAGGACTCGTTTGTACCCGTTGATGATAGAATGGG) were diluted 1/10 five times to measure the content of telomeric sequence per sample in kb and diploid genome copies per sample, respectively (O’Callaghan and Fenech, 2011).

Each PCR reaction was performed in triplicate and three non-template controls were included on each 96-well plate. All samples were analyzed on the Roche LightCycler^®^ 480. Telomere PCR conditions were: one cycle at 95°C for 15 min, followed by 40 cycles of 95°C for 10 s, 57°C for 10 s and acquisition at 72°C for 10 s, followed by a melting curve: one cycle of 95°C for 5 s, 65°C for 30 s and 97°C continuous. Finally, a cooling step: 40°C for 30 sec. The single-copy genome 36B4 PCR conditions were: one cycle for 15 min at 95°C; followed by 40 cycles of 95°C for 10 s, 60°C for 10 s with 10 s at 72°C with signal acquisition; followed by a melting curve of one cycle of 95°C for 5 s, 65°C for 30 s and 97°C continuous. The final step consisted of cooling at 40°C for 30 s. After amplification, raw data was generated using LightCycler^®^ 480 software version 1.5.1.62 SP3. Raw ct values were transformed into Kb and diploid genome copies per sample using the linear formula generated from each standard curve (*r*^2^ = 0.99, for both curves). T/S ratio (absolute quantification divided by absolute telomere length) was calculated by dividing Kb data into diploid genome copies (O’Callaghan and Fenech, 2011).

#### Covariates: Socioeconomics and maternal mood

Mothers reported their current age, family income, education, and depression levels at the 36-months and 7-years post-partum visits. Depression levels were assessed using the Center for Epidemiologic Studies Depression Scale (CES-D; Radolf, 1977). The CES-D scores can vary from zero to 60 and a cut-off of 16 is used for a clinical diagnosis of depression (Radolf, 1977).

### Data analysis

Descriptive statistics and inter-correlations were computed for all the variables in the analyses. Biochemical measures were summarized and their distributions investigated. Cortisol values across measurements and TL were positively skewed. Two participants had cortisol levels above 4 standard deviations from the mean and were removed from the analysis. The remaining values were log10 transformed, resulting in normal distributions for all samples. Generalized estimating equation models were fit with an autoregressive correlation structure to take into account the large correlations of time adjacent measurements, and a systematically decreasing correlation with increasing distance measurements when estimating standard errors (Fitzmaurice et al., 2011). We restricted the model to include terms of theoretical interest such as main effects of the repeated cortisol measurements (time), attachment styles, maternal education, sex, and family income. We also included a term for two-way interactions (attachment × income; and attachment × time) but this term did not reach statistical significance and therefore it was dropped. At this stage, the *geepack* R (version 4.0.2) package was used (Halekoh et al., 2006).

We examined cognitive neurodevelopment differences across attachment groups using univariate analysis of covariance (ANCOVA) concurrently controlling for sex, income, maternal education, and depression level, at this stage we only included the significant covariates in the models, for those models we reported the associated Betas (B), Cohen’s r equivalente effect size (Rosenthal and Rubin, 2003), and *p*-values. Finally, mediation analyses were performed to examine attachment style effects on child neurodevelopment via cortisol response. At this stage, cortisol measurements were aggregated using area under the curve with respect to the ground (*AUC*_g_; Pruessner et al., 2003) to serve as a predictor and mediator. *AUC*_g_ accounts for baseline cortisol levels and the change associated with posterior measurements by capturing the distance of measurements in relation the minimum detected value. For mediation analysis, we followed standard procedures suggested by Jose (2013) in which mediation parameter estimates, standard errors, and confidence bounds for total and indirect effects were obtained by bootstrapping 10,000 bias-corrected samples using the *lavaan* package (Rosseel, 2012). Ninety-five percent bootstrap confidence intervals were reported in order to achieve robust non-parametric inference. Bootstrapped confidence intervals that do not include zero indicate that the independent variable has an indirect effect on the dependent variable via the mediator (Bollen and Stine, 1990). For TL analysis at age seven, we conducted three ANCOVAs with TL as the dependent variable: a model with an interaction between attachment and *AUC*_g_ cortisol, a model with an interaction between attachment and age at TL sample collection, and a model with the main effects of *AUC*_g_ cortisol and attachment. At this stage, we also screened for significant covariates.

## Results

At age three the sample sizes for the attachment groups were 61 for secure and 46 for insecure children, at age seven 40, and 32, respectively. In all analysis attachment classification (0 = secure, 1 = insecure) and sex (0 = male, 1 = female) were dummy coded. Table 1 shows the descriptive statistics and significance levels of the Student’s *t*-test for all the measures analyzed by attachment style. Children from the insecure attachment group had poorer neurodevelopmental scores (BSID, *p* = 0.02 for 3 years old and *p* = 0.04 for 7 years old) and a higher aggregated cortisol *AUC*_g_ (*p* = 0.033) at age three. Mothers of children with different attachment styles showed no significant differences concerning education, family income, and the CES-D depression score for both ages of children.

**TABLE 1:** Sample demographics and study variables descriptive statistics.

Variables	3 years sample (*N* = 107)				7 years sample (*N* = 71)			
	Secure	Insecure	*p*	Cohen’s *d*	Secure	Insecure	*p*	Cohen’s *d*
						
	M or N (SD or %)	M or N (SD or %)			M or N (SD or %)	M or N (SD or %)		
Maternal education^1^			0.352	0.18			0.798	−0.06
High school diploma or less	3 (4.92)	3 (6.52)			4 (10.26)	6 (18.75)		
Some community college	4 (6.56)	7 (15.22)			0 (0)	0 (0)		
Complete community college or university	21 (34.43)	13 (28.26)			16 (41.03)	7 (21.88)		
University degree	33 (54.1)	23 (50)			19 (48.72)	19 (59.38)		
Family income^1^			0.108	0.32			0.325	−0.24
15,000 or less	2 (3.28)	2 (4.35)			0 (0.00)	2 (6.45)		
Between $15,000 and $40,000	7 (11.48)	14 (30.43)			6 (15.38)	5 (16.13)		
Between $40,000 and $80,000	19 (31.15)	12 (26.09)			11 (28.21)	9 (29.03)		
Between $80,000 and $100,000	33 (54.1)	18 (39.13)			22 (56.41)	15 (48.39)		
CESD depression score	10.43 (10.15)	12.85 (10.01)	0.225	0.24	9.87 (11.19)	13.19 (11.19)	0.209	0.31
BSID neurodevelopment score	102.55 (8.85)	97.32 (13.67)	0.02	0.47	103.87 (6.76)	98.38 (6.76)	0.038	−0.51
Cortisol 20-min pre	4.2 (5.57)	4.85 (5.05)	0.538	0.12	4.58 (6.9)	4.7 (6.9)	0.93	0.02
Cortisol 20 min post	3.41 (4.26)	3.66 (1.83)	0.719	0.07	3.78 (5.28)	3.49 (5.28)	0.766	−0.07
Cortisol 40 min post	3.58 (3.36)	4.08 (2.67)	0.406	0.16	3.98 (4.09)	4.39 (4.09)	0.64	0.11
Cortisol 60 min post	3.56 (1.97)	4.23 (2.24)	0.104	0.32	3.79 (2.25)	4.41 (2.25)	0.264	0.27
Cortisol *AUC*_g_^2^	5.53 (0.05)	5.72 (0.06)	0.033	0.43	5.58 (0.07)	5.72 (0.08)	0.214	0.31

*P* values are based on independent samples Student’s *t* tests.

^1^Variables were categorized for sake of description.

^2^Mean-adjusted values for maternal education and household income, values in parenthesis depicts standard errors. Cohen’s d: Cohen (1992) measure of effect size.

The inter-correlations between the studied variables are reported in Table 2. Cortisol levels were significantly intercorrelated, *p*’s < 0.001, and Levene’s test indicated homogeneous variance across attachment groups: 20-min pre (*F* = 2.06, *p* = 0.15), 20-min post (*F* = 0.36, *p* = 0.55), 40-min post (*F* = 0.08, *p* = 0.78), 60-min post (*F* = 0.51, *p* = 0.47), and *AUC*_*g*_ (*F* = 0.06, *p* = 0.81). Maternal education, income, and CES-D scores also presented significant correlations. Children who scored higher on the BSID scale also had mothers with a higher education level (*p* < 0.05). TL was significantly correlated with children’s age at the 7-years visit (*r* = −0.33, *p* = 0.005).

**TABLE 2:** Descriptive statistics and correlations among variables.

		M (SD)	1	2	3	4	5	6	7	8	9
1	Maternal education	3.31 (0.87)	–								
2	Family income	13.96 (3.68)	0.40**	–							
3	CESD depression score	11.48 (10.11)	−0.22*	−0.28*	–						
4	BSID neurodevelopment score	100.34 (11.39)	0.27*	0.07	–0.15	–					
5	Cortisol 20-min pre	4.48 (5.34)	0.08	0.12	–0.01	0.08	–				
6	Cortisol 20 min post	3.52 (3.42)	0.06	0.06	0.00	0.05	0.86**	–			
7	Cortisol 40 min post	3.80 (3.08)	0.08	0.03	–0.11	0.09	0.59**	0.65**	–		
8	Cortisol 60 min post	3.85 (2.11)	0.02	0.02	–0.07	0.10	0.45**	0.46**	0.75**	–	
9	Cortisol *AUC*_g_	2.32 (0.43)	0.03	0.03	–0.05	0.06	0.78**	0.75**	0.80**	0.74**	–
10	Telomere length	5.43 (0.57)	0.00	–0.05	–0.16	0.21	0.05	0.05	–0.03	–0.05	0.07

Family income was measured by a 17-point scale ranging from 0 (“no revenue”) to 17 (“at least 100,000 Can$ per year”).

Maternal education was measured by a four-point scale (1 = “high school diploma or less”; 2 = “some community college”; 3 = “completed community college or some university”; and 4 = “University degree or higher”).

CESD, Center for Epidemiologic Studies Depression Scale; BSID, Bayley Scale of Infant Development; *AUC_g_*, ground Area Under the Curve.

*p < 0.05, **p < 0.001.

**FIGURE 1:**
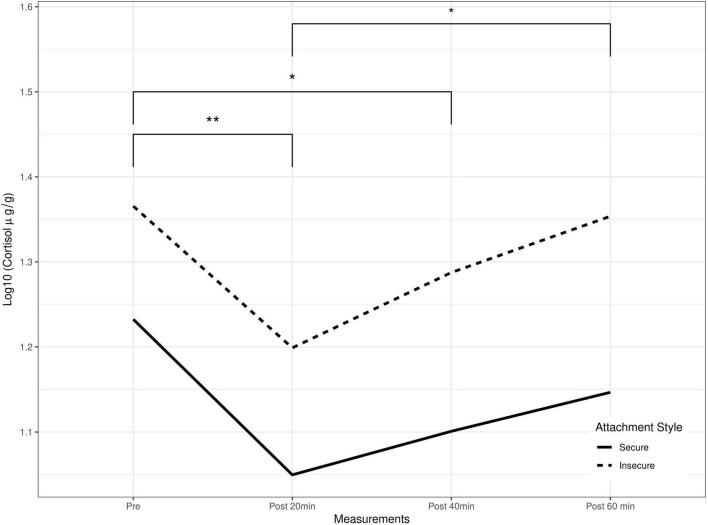
Salivary cortisol levels as a function of time and attachment style. GEE’s predicted cortisol as a function of attachment styles and repeated measures, *p < 0.05, **p < 0.01.

The investigation of repeated cortisol measurements using GEE modeling with attachment style, child’s sex, maternal education, and income level yielded a significant prediction of cortisol response (Table 3). Children with an insecure attachment to their caregiver had higher general cortisol production compared to those with a secure attachment (Figure 1).

**TABLE 3:** GEE model of cortisol response before and after the Strange Situation Experiment.

	B	SE	95% CI		*χ^2^*
			Lower	Upper	
Attachment = insecure	0.17*	0.08	0.01	0.31	4.81
**Cortisol measurement**					
20-min pre vs. 20-min post	−0.17**	0.04	–0.24	–0.09	21.66
20-min pre vs. 40-min post	−0.11*	0.05	–0.20	–0.01	4.96
20-min pre vs. 60-min post	–0.05	0.05	–0.15	0.05	1.07

χ^2^ = Wald’s chi-square.

*p < 0.05, **p < 0.001.

Pairwise repeated cortisol measures using Tukey correction indicated significant differences between the 20-min before the experiment in comparison to the 20-min post the strange situation, and between the post-20-min and post-40-min measurements. None of the covariates had a significant association with the outcome (Table 3 and Figure 1).

**FIGURE 2:**
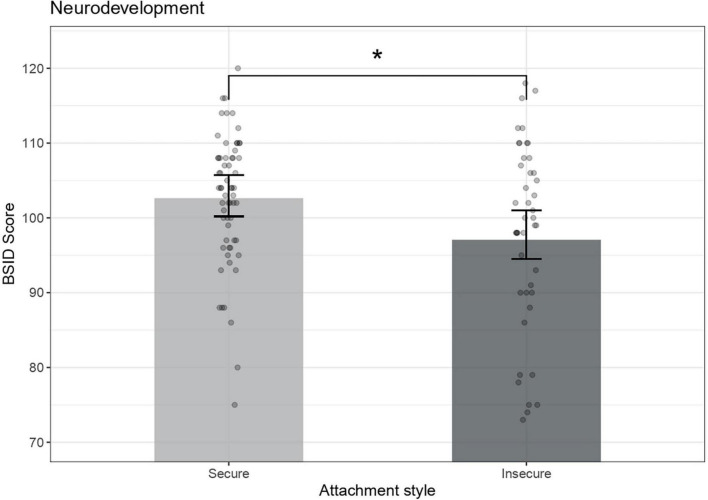
Preschoolers’ neurodevelopment across attachment groups. Bayle Scales of Infant Development mental scores are adjusted for child’s sex [*F* (1,96) = 7.76, *p* = 0.023]. Error bars represent 95% confidence intervals of the means, *p < 0.05.

ANCOVA results indicated that children with insecure attachment had poorer neurodevelopmental scores in comparison with children from the secure group (*B* = −5.17, *r* = 0.23, *p* = 0.017). Child’s sex (B _Female_ = 4.93, *r* = 0.22, *p* = 0.023) was also significantly associated with BSID scores (Figure 2).

**FIGURE 3:**
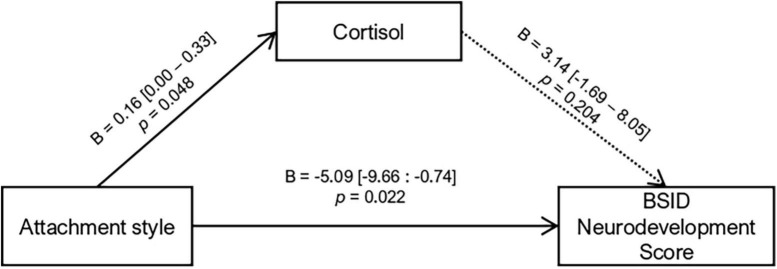
Mediation analysis for Attachment Style effects on child neurodevelopment via cortisol *AUC_*g*_*. Total effects: *B* = –4.58 [–8.66: –0.30], *p* = 0.032; indirect effects: *B* = 0.52 [–0.12: 2.40], *p* = 0.365. Values inside brackets indicate 95% bootstrapped confidence interval.

Since attachment styles were associated with cortisol *AUC*_*g*_ and BSID scores, we proceeded to investigate if the association of attachment and neurodevelopment was mediated by cortisol *AUC_*g*_.* The mediation model indicated a direct main effect of attachment style on cortisol *AUC*_*g*_ (*p* = 0.048) and neurodevelopment (*p* = 0.022). The association between attachment style and neurodevelopment was not mediated by cortisol (indirect *B* = 0.52, *p* = 0.365) nor interacted (*B* = 4.17, *p* = 0.409), indicating that attachment style was independently associated with the aggregated levels of cortisol and neurodevelopment (see Figure 3 for the estimated parameters of the mediation model).

**FIGURE 4:**
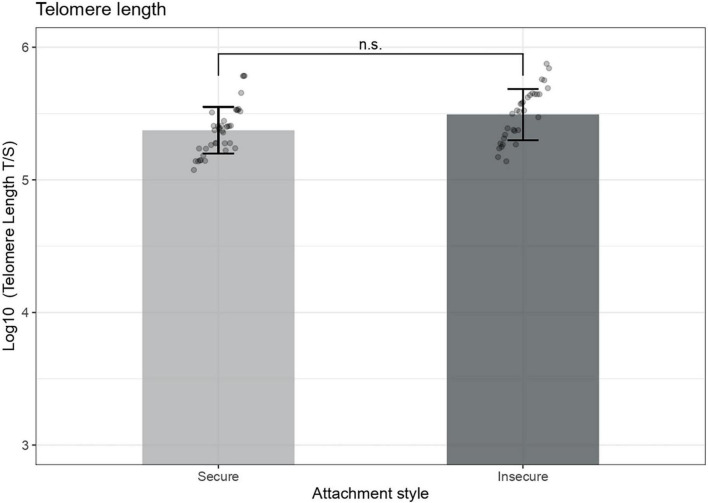
Preschooler’s telomere length across attachment groups. Telomere length measures are adjusted for child’s age at sample collection, *F* (1,68) = 8.29, *p* = 0.005. Error bars represent 95% confidence intervals of the means, *n.s.*, Not significant.

We then explored whether there was a difference between attachment groups in the telomere length in our follow-up sample. ANCOVA results indicated that there were no significant interaction between cortisol *AUC*_*g*_ and attachment [*F* (1,66) = 0.32, *B* = −0.175, *p* = 0.569], attachment and age [*F* (1,66) = 0.03, *B* = −0.02, *p* = 0.857], nor significant differences in telomere length of 7-years-olds across attachment groups, *F* (1,66) = 0.81, *p* = 0.368 (Figure 4). The only covariate that showed a significant association with telomere length was children’s age at sample collection (*B* = −0.13, Cohen’s *r* equivalent = 0.33, *p* = 0.005). Maternal education, income, and CES-D score at age seven were not significantly associated with telomere length.

## Discussion

Insecure attachment styles in infancy are associated with a variety of negative developmental outcomes, including poor mental and cognitive functions such as intelligence executive functioning, physical health, and atypical social behavior (Colonnesi et al., 2011; Maras et al., 2016; Fong et al., 2017). Alterations in the development of stress response systems are thought to be a mediating mechanism linking adverse experiences to the onset of developmental problems (McLaughlin et al., 2015). The findings of the present study extend to the preschool period evidence of the importance of attachment since, compared with the vast infant literature, relatively few studies have examined associations of preschool attachment with cortisol secretion and neurodevelopment (Moss et al., 2004). Our results also highlight that at age three a secure bond between the child and the caregiver is associated with reduced physiological cortisol activity elicited by the emotional experience probed by the Strange Situation Experiment. At the behavioral level, the insecure attachment was significantly associated with poorer neurodevelopment BSID scores, highlighting the importance of early child-caregiver interactions for the attainment of early cognitive abilities that are predictive of complex cognitive function and learning in later development (Krogh and Væver, 2019; Rubio-Codina and Grantham-McGregor, 2020).

Mediation analysis did not provide evidence for the role of salivary cortisol response in mediating the association between attachment style and neurodevelopment, suggesting that the effect of insecure attachment on poor neurodevelopment task performance and higher levels of overall cortisol were independent. Those findings are in line with the notion that the attachment relationship between children and caregivers is a necessary scaffold for the development of numerous skills, including emotion regulation, language, and school readiness (Rispoli et al., 2013; Cooke et al., 2016). The direct association of attachment styles with cognitive neurodevelopment might be explained by the fact that a secure and stable bond facilitates the observation of parental behavior by the child favoring modeling learning (Bjorklund, 2012). Another aspect of children’s learning is that parental presence changes the nature and content of associative learning processes by facilitating exploration, indicating that learning systems during early childhood are developed to allow modulation as a function of parental presence (Tottenham et al., 2019). Taken together, findings regarding HPA axis reactivity, neurodevelopment, and attachment security emphasize the relevance of the early relationship to shaping development at biological and behavioral levels. Thus, parents’ efforts toward establishing mutually engaging and emotionally available interactions with their children might improve socio-emotional and cognitive functioning in the early years (Rispoli et al., 2013; Reilly and Gunnar, 2019).

It is worth noting that we took advantage of a well-established experimental procedure to determine the pattern of the child-caregiver bond and measured the physiological HPA axis response immediately before and after testing. Since the Strange Situation Experiment is, in many cases, a stress-invoking experience (Ainsworth et al., 2015), the HPA axis response measurement immediately before and after the procedure allowed us to rely on objective measures that control for bias and social desirability effects present on self-report measures (Fisher and Katz, 2000). Interestingly, we observed a decrease 20 min after the experiment, followed by an increase at the 40- and 60-min measurements for both groups. Although an immediate increase would be expected, non-significant cortisol changes in relation to challenging exposures have been reported previously (Roque et al., 2012). It is possible that the continued assessment of cortisol levels after 60 min of the experiment could auxiliate in the distinction of HPA functioning as a function of attachment styles. Additionally, previous studies that assessed attachment style and cortisol response before and after the Strange Situation Experiment in infants also observed an association with insecure attachment yielding higher cortisol *AUC*_g_, and reactivity (Van Bakel and Riksen-Walraven, 2004). Although Van Bakel and Riksen-Walraven (2004) found evidence of interaction between cortisol response and attachment, in which insecure infants showed variable cognitive competence as a function of cortisol reactivity, this pattern of association was not replicated in our sample of preschoolers. Nonetheless, an insecure attachment was associated with poorer neurodevelopment and higher levels of cortisol response in line with the notion that non-secure attachment may affect several mechanisms, placing children on a path to future psychopathology (Fong et al., 2017; Nofech-Mozes et al., 2019).

Cortisol levels and attachment group, as well as their interaction, were not correlated with TL at 7y. Although we used repeated measures of cortisol compiled in the form of the *AUC*_*g*_, salivary cortisol might be too dynamic to capture long-term HPA axis activity (Flom et al., 2017). Accordingly, a meta-analysis that considered basal cortisol or cortisol reactivity in cross-sectional studies did not observe a significant association with TL (Jiang et al., 2019). Conversely, Barha et al. (2017) found that morning urinary cortisol assessed over 7 weeks was negatively associated with TL, indicating that a longer cortisol monitoring could be better suited for capturing chronic exposures. Therefore, another marker of HPA function, hair cortisol concentration, might be a more appropriate marker of cumulative/chronic cortisol exposure (Flom et al., 2017). Additionally, we acknowledge that an initial measure of TL could inform on individual variation in the rate of TL shortening (Daniali et al., 2013). However, this information is not available at age 3 in our sample. One could speculate based on previous findings that TL at birth and age-dependent shortening thereafter are highly variable across individuals (Benetos et al., 2019), and as a measure associated with individual variation, attachment might contribute to the trajectory of this biomarker. Some findings indicate that individual variation in the rate of TL in adulthood is usually insufficient to overcome interindividual variation in TL in pre-adulthood years – therefore, individuals with comparatively short or long TL typically maintain a similar length (short or long in comparison to its peers) throughout their remaining life course (O’Callaghan and Fenech, 2011). In contrast, prenatal adversity (like smoking during pregnancy) is associated with greater TL shortening from four to 18 months of age (Howell et al., 2022). Thus, since in many cases attachment is viewed as buffer to adversity, the examination of TL trajectories warrants future studies targeting attachment, neurodevelopment, and the HPA axis response.

Although our study considered repeated measures of cortisol, gold-standard tests, and experimental procedures, some limitations and future directions should be considered. First, the sample size used in this study was relatively small and attrition of 33.6% was observed for the data collection at the year-seven visit. A larger sample would allow differentiating insecure attachment groups into insecure-avoidant, insecure-ambivalent/dependent, and insecure-disorganized subgroups. A more representative sample from the disorganized group could lead to stronger identifiable associations with TL since children exhibiting an insecure-disorganized attachment style appear to be at greater risk for dysfunctional outcomes and genome-wide DNA methylation (Fearon et al., 2010; Garg et al., 2018). Nevertheless, the combination of a general insecure attachment subtype is extensively acknowledged as a risk factor grounded by previous work (e.g., Van Bakel and Riksen-Walraven, 2004; O’Connor and McCartney, 2007; Roque et al., 2012; Rispoli et al., 2013; Bernier et al., 2015; Johnson et al., 2018). Additionally, the replication of our findings with larger samples with varying levels of risk is relevant since our results are based on a relatively well-schooled and low-risk sample. This is in line with large evidence that has shown that attachment is a moderator of the effects of early life adversity, such as poverty, exposure to violence, and neglect (Siqueira et al., 2006; McLaughlin et al., 2015; Dagan et al., 2018; Johnson et al., 2018; Measelle and Ablow, 2018).

In summary, the findings of the present study extend to the preschool period evidence to the existing literature on the detrimental effects of insecure attachment as an aggravator of the physiological effects of stress with the increase of cortisol release associated with the Strange Situation Experiment emotional reaction. At the behavioral level, the insecure attachment was significantly associated with poorer neurodevelopment BSID scores, emphasizing the importance of early child-caregiver interactions for the attainment of early cognitive abilities that are predictive of complex cognitive function and learning in later development. The associations of insecure attachment with poor neurodevelopment and higher levels of overall cortisol highlight the importance of this precursor of early social development. Although we did not observe a significant association with TL, prevention and intervention policies that promote secure attachment patterns in early life are more likely to obtain promising effects on several health domains (Dozier et al., 2008; Newman et al., 2015; Julian et al., 2018). A wider understanding of the consequences of the attachment behavioral system is expected to ground caregiving practices and help to inform health and educational settings at multiple levels.

## Data availability statement

The data analyzed in this study is subject to the following licenses/restrictions: The data that support the findings of this study are available on reasonable request from the corresponding author, PPS. The data are not publicly available due to information that could compromise the privacy of research participants. Requests to access these datasets should be directed to PPS, patricia.silveira@mcgill.ca.

## Ethics statement

The studies involving human participants were reviewed and approved by the McGill University St-Joseph’s Hospital. Written informed consent to participate in this study was provided by the participants’ legal guardian/next of kin.

## Author contributions

EJMF was involved in data analysis, preparation, and review of the manuscript. AF was involved in cortisol data curation and review of the manuscript. IP, DA, and BB were involved in preparation, data analysis interpretation, and review of the manuscript. C-AT was involved in telomere length processing. RS, AW, LA, MM, and PPS were involved in conception and review of the manuscript. All authors contributed to the article and approved the submitted version.

## Footnotes

1
https://salimetrics.com/wp-content/uploads/2018/03/salivary-cortisol-elisa-kit.pdf

## References

[B1] Ainsworth M. D. S. Blehar M. C. Waters E. Wall S. N. (2015). *Patterns of Attachment: a Psychological Study of the Strange Situation.* New York, NY: Routledge.

[B2] Alvarenga P. Cerezo M. Á Wiese E. Piccinini C. A. (2019). Effects of a short video feedback intervention on enhancing maternal sensitivity and infant development in low-income families. *Attach. Hum. Dev.* 22 534–554. 10.1080/14616734.2019.1602660 30961424

[B3] Ashman S. B. Dawson G. Panagiotides H. Yamada E. Wilkinson C. W. (2002). Stress hormone levels of children of depressed mothers. *Dev. Psychopathol.* 14 333–349. 10.1017/s0954579402002080 12030695

[B4] Badanes L. S. Dmitrieva J. Watamura S. E. (2012). Understanding cortisol reactivity across the day at child care: The potential buffering role of secure attachments to caregivers. *Early Child. Res. Q.* 27 156–165. 10.1016/j.ecresq.2011.05.005 22408288 PMC3295236

[B5] Barha C. K. Salvante K. G. Hanna C. W. Wilson S. L. Robinson W. P. Altman R. M. (2017). Child mortality, hypothalamic-pituitaryadrenal axis activity and cellular aging in mothers. *PLoS One* 12:e0177869. 10.1371/journal.pone.0177869 28542264 PMC5444612

[B6] Bayley N. (1993). *Bayley Scales of Infant Development*, 2nd Edn. San Antonio, TX: The Psychological Corporation.

[B7] Benetos A. Verhulst S. Labat C. Lai T. P. Girerd N. Toupance S. (2019). Telomere length tracking in children and their parents: Implications for adult onset diseases. *FASEB J.* 33 14248–14253. 10.1096/fj.201901275R 31652401 PMC6894096

[B8] Bergman K. Sarkar P. Glover V. O’Connor T. G. (2008). Quality of child-parent attachment moderates the impact of antenatal stress on child fearfulness. *J. Child Psychol. Psychiatry* 49 1089–1098. 10.1111/j.1469-7610.2008.01987.x 19017025 PMC2673696

[B9] Bernier A. Beauchamp M. H. Carlson S. M. Lalonde G. (2015). A secure base from which to regulate: Attachment security in toddlerhood as a predictor of executive functioning at school entry. *Dev. Psychol.* 51 1177–1189. 10.1037/dev0000032 26192039

[B10] Bjorklund D. F. (2012). *Children’s Thinking: Cognitive Development and Individual Differences*, 5th Edn. Belmont, CA:Cengage Learning.

[B11] Bollen K. A. Stine R. (1990). Direct and indirect effects: Classical and bootstrap estimates of variability. *Sociol. Methodol.* 20:115. 10.2307/271084

[B12] Bowlby J. (1988). *A Secure Base: Parent-Child Attachment and Healthy Human Development.* New York: Basic Books.

[B13] Cassidy J. (2016). “The Nature of the Child’s Ties,” in *Handbook of Attachment?: Theory, Research, and Clinical Applications*, ed. Cassidy J. (New York, NY: The Guilford Press), 3–24.

[B14] Cawthon R. M. (2009). Telomere length measurement by a novel monochrome multiplex quantitative PCR method. *Nucleic Acids Res.* 37:e21. 10.1093/nar/gkn1027 19129229 PMC2647324

[B15] Cohen J. (1992). A power primer. *Psychol. Bull.* 112, 155–159. 10.1037/0033-2909.112.1.155 19565683

[B16] Colonnesi C. Draijer E. M. Stams G. J. J. M. van der Bruggen C. O. Bögels S. M. Noom M. J. (2011). The relation between insecure attachment and child anxiety: A meta-analytic review. *J. Clin. Child Adolesc. Psychol.* 40 630–645. 10.1080/15374416.2011.581623 21722034

[B17] Cooke J. E. Stuart-Parrigon K. L. Movahed-Abtahi M. Koehn A. J. Kerns K. A. (2016). Children’s emotion understanding and mother–child attachment: A meta-analysis. *Emotion* 16 1102–1106. 10.1037/emo0000221 27606827

[B18] Crittenden P. M. (1992). Quality of attachment in the preschool years. *Dev. Psychopathol.* 4 209–241. 10.1017/S0954579400000110

[B19] Da Silva M. A. de Mendonça Filho E. J. Mônego B. G. Bandeira D. R. (2018). Instruments for multidimensional assessment of child development: A systematic review. *Early Child Dev. Care* 190 1257–1271. 10.1080/03004430.2018.1528243

[B20] Dagan O. Asok A. Steele H. Steele M. Bernard K. (2018). Attachment security moderates the link between adverse childhood experiences and cellular aging. *Dev. Psychopathol.* 30 1211–1223. 10.1017/S0954579417001705 29229013

[B21] Daniali L. Benetos A. Susser E. Kark J. D. Labat C. Kimura M. (2013). Telomeres shorten at equivalent rates in somatic tissues of adults. *Nat. Commun.* 4:1597. 10.1038/ncomms2602 23511462 PMC3615479

[B22] Dozier M. Peloso E. Lewis E. Laurenceau J. P. Levine S. (2008). Effects of an attachment-based intervention on the cortisol production of infants and toddlers in foster care. *Dev. Psychopathol.* 20 845–859. 10.1017/S0954579408000400 18606034 PMC3258505

[B23] Fearon R. P. Bakermans-Kranenburg M. J. van IJzendoorn M. H. Lapsley A. M. Roisman G. I. (2010). The significance of insecure attachment and disorganization in the development of children’s externalizing behavior: A meta-analytic study. *Child Dev.* 81 435–456. 10.1111/j.1467-8624.2009.01405.x 20438450

[B24] Finzi R. Ram A. Har-Even D. Shnit D. Weizman A. (2001). Attachment styles and aggression in physically abused and neglected children. *J. Youth Adolesc.* 30 769–786. 10.1023/A:1012237813771

[B25] Fisher R. J. Katz J. E. (2000). Social-desirability bias and the validity of self-reported values. *Psychol. Mark.* 17 105–120.

[B26] Fitzmaurice M. G. Laird N. M. Ware J. H. (2011). *Applied Longitudinal Analysis*, 2th Edn. Hoboken, NJ: Wiley.

[B27] Flom M. John A. M. St Meyer J. S. Tarullo A. R. (2017). Infant hair cortisol: Associations with salivary cortisol and environmental context. *Dev. Psychobiol.* 59 26–38. 10.1002/dev.21449 27472986 PMC6203946

[B28] Fong M. C. Measelle J. Conradt E. Ablow J. C. (2017). Links between early baseline cortisol, attachment classification, and problem behaviors: A test of differential susceptibility versus diathesis-stress. *Infant Behav. Dev.* 46 158–168. 10.1016/j.infbeh.2017.01.005 28171802 PMC5546087

[B29] Garg E. Chen L. Nguyen T. T. T. Pokhvisneva I. Chen L. M. Unternaehrer E. (2018). The early care environment and DNA methylome variation in childhood. *Dev. Psychopathol.* 30 891–903. 10.1017/S0954579418000627 30068421

[B30] Gervai J. (2009). Environmental and genetic influences on early attachment. *Child Adolesc. Psychiatry Ment. Health* 3:25. 10.1186/1753-2000-3-25 19732441 PMC2753321

[B31] Gotlib I. H. Lemoult J. Colich N. L. Foland-Ross L. C. Hallmayer J. Joormann J. (2015). Telomere length and cortisol reactivity in children of depressed mothers. *Mol. Psychiatry* 20 615–620. 10.1038/mp.2014.119 25266121 PMC4419149

[B32] Halekoh U. Højsgaard S. Yan J. (2006). The R package geepack for generalized estimating equations. *J. Stat. Softw.* 15 1–11. 10.18637/jss.v015.i02

[B33] Handley E. D. Michl-Petzing L. C. Rogosch F. A. Cicchetti D. Toth S. L. (2017). Developmental cascade effects of interpersonal psychotherapy for depressed mothers: Longitudinal associations with toddler attachment, temperament, and maternal parenting efficacy. *Dev. Psychopathol.* 29 601–615. 10.1017/S0954579417000219 28401849 PMC5407290

[B34] Houben J. M. J. Moonen H. J. J. van Schooten F. J. Hageman G. J. (2008). Telomere length assessment: Biomarker of chronic oxidative stress? *Free Radic. Biol. Med.* 44 235–246. 10.1016/j.freeradbiomed.2007.10.001 18021748

[B35] Houbrechts M. Cuyvers B. Goossens L. Bijttebier P. Bröhl A. S. Calders F. (2021). Parental support and insecure attachment development: the cortisol stress response as a moderator. *Attach. Hum. Dev.* [Epub ahead of print]. 10.1080/14616734.2021.1907968 33871320

[B36] Houdé O. Rossi S. Lubin A. Joliot M. (2010). Mapping numerical processing, reading, and executive functions in the developing brain: An fMRI meta-analysis of 52 studies including 842 children. *Dev. Sci.* 13 876–885. 10.1111/j.1467-7687.2009.00938.x 20977558

[B37] Howell M. P. Jones C. W. Herman C. A. Mayne C. V. Fernandez C. Theall K. P. (2022). Impact of prenatal tobacco smoking on infant telomere length trajectory and ADHD symptoms at 18 months: A longitudinal cohort study. *BMC Med.* 20:153. 10.1186/s12916-022-02340-1 35477473 PMC9047258

[B38] IJzendoorn M. H. Dijkstra J. Bus A. G. (1995). Attachment, intelligence, and language: A Meta-analysis. *Soc. Dev.* 4 115–128. 10.1111/j.1467-9507.1995.tb00055.x

[B39] Jiang Y. Da W. Qiao S. Zhang Q. Li X. Ivey G. (2019). Basal cortisol, cortisol reactivity, and telomere length: A systematic review and meta-analysis. *Psychoneuroendocrinology* 103 163–172. 10.1016/j.psyneuen.2019.01.022 30695740 PMC6450740

[B40] Johnson A. B. Mliner S. B. Depasquale C. E. Troy M. Gunnar M. R. (2018). Attachment security buffers the HPA axis of toddlers growing up in poverty or near poverty: Assessment during pediatric well-child exams with inoculations. *Psychoneuroendocrinology* 95 120–127. 10.1016/j.psyneuen.2018.05.030 29852405

[B41] Jose P. E. (2013). *Doing Statistical Mediation and Moderation.* London: The Guilford Press.

[B42] Julian M. M. McCall R. B. Groark C. J. Muhamedrahimov R. J. Palmov O. I. Nikiforova N. V. (2018). Development of children adopted to the United States following a social–emotional intervention in St. Petersburg (Russian Federation) institutions. *Appl. Dev. Sci.* 23:273-293. 10.1080/10888691.2017.1420480 31488944 PMC6727650

[B43] Krogh M. T. Væver M. S. (2019). A longitudinal study of the predictive validity of the Bayley-III scales and subtests. *Eur. J. Dev. Psychol.* 16 727–738. 10.1080/17405629.2018.1485563

[B44] Kudielka B. M. Buske-Kirschbaum A. Hellhammer D. H. Kirschbaum C. (2004). HPA axis responses to laboratory psychosocial stress in healthy elderly adults, younger adults, and children: Impact of age and gender. *Psychoneuroendocrinology* 29 83–98. 10.1016/S0306-4530(02)00146-4 14575731

[B45] Kudielka B. M. Kirschbaum C. (2005). Sex differences in HPA axis responses to stress: A review. *Biol. Psychol.* 69 113–132. 10.1016/j.biopsycho.2004.11.009 15740829

[B46] Maras D. Obeid N. Flament M. Buchholz A. Henderson K. A. Gick M. (2016). Attachment style and obesity: Disordered eating behaviors as a mediator in a community sample of canadian youth. *J. Dev. Behav. Pediatr.* 37 762–770. 10.1097/DBP.0000000000000361 27801724

[B47] Martens D. S. Janssen B. G. Bijnens E. M. Clemente D. B. P. Vineis P. Plusquin M. (2020). Association of Parental Socioeconomic Status and Newborn Telomere Length. *JAMA Netw. Open* 3:e204057. 10.1001/jamanetworkopen.2020.4057 32364595 PMC7199116

[B48] McEwen B. S. Gianaros P. J. (2010). Central role of the brain in stress and adaptation: Links to socioeconomic status, health, and disease. *Ann. N.Y. Acad. Sci.* 1186 190–222. 10.1111/j.1749-6632.2009.05331.x 20201874 PMC2864527

[B49] McLaughlin K. A. Sheridan M. A. Tibu F. Fox N. A. Zeanah C. H. Nelson C. A. (2015). Causal effects of the early caregiving environment on development of stress response systems in children. *Proc. Natl. Acad. Sci. U.S.A.* 112 5637–5642. 10.1073/pnas.1423363112 25902515 PMC4426436

[B50] Meaney M. J. Szyf M. (2005). Environmental programming of stress responses through DNA methylation: Life at the interface between a dynamic environment and a fixed genome. *Dialogues Clin. Neurosci.* 7 103–123. 10.31887/DCNS.2005.7.2/mmeaney 16262207 PMC3181727

[B51] Measelle J. R. Ablow J. C. (2018). Contributions of early adversity to pro-inflammatory phenotype in infancy: The buffer provided by attachment security. *Attach. Hum. Dev.* 20 1–23. 10.1080/14616734.2017.1362657 28797194

[B52] Menon M. Katz R. C. Easterbrooks M. A. (2020). Linking Attachment and Executive Function Systems: Exploring Associations in a Sample of Children of Young Mothers. *J. Child Fam. Stud.* 29 2314–2329. 10.1007/s10826-020-01759-5

[B53] Modabbernia A. Reichenberg A. Ing A. Moser D. A. Doucet G. E. Artiges E. (2020). Linked patterns of biological and environmental covariation with brain structure in adolescence: A population-based longitudinal study. *Mol. Psychiatry* 26 4905–4918. 10.1038/s41380-020-0757-x 32444868 PMC7981783

[B54] Moss E. Bureau J. F. Cyr C. Mongeau C. St-Laurent D. (2004). Correlates of attachment at age 3: Construct validity of the preschool attachment classification system. *Dev. Psychol.* 40 323–334. 10.1037/0012-1649.40.3.323 15122960

[B55] Moss E. Cyr C. Bureau J. F. Tarabulsy G. M. Dubois-Comtois K. (2005). Stability of attachment during the preschool period. *Dev. Psychol.* 41 773–783. 10.1037/0012-1649.41.5.773 16173874

[B56] Nelson C. A. Gabard-Durnam L. J. (2020). Early adversity and critical periods: Neurodevelopmental consequences of violating the expectable environment. *Trends Neurosci.* 43 133–143. 10.1016/j.tins.2020.01.002 32101708 PMC8092448

[B57] Newman L. Sivaratnam C. Komiti A. (2015). Attachment and early brain development – neuroprotective interventions in infant–caregiver therapy. *Transl. Dev. Psychiatry* 3:28647. 10.3402/tdp.v3.28647

[B58] Nofech-Mozes J. Pereira J. Gonzalez A. Atkinson L. (2019). Cortisol secretion moderates the association between mother–infant attachment at 17 months and child behavior at age 5 years. *Dev. Psychobiol.* 61 239–253. 10.1002/dev.21799 30446993

[B59] O’Callaghan N. J. Fenech M. (2011). A quantitative PCR method for measuring absolute telomere length. *Biol. Proced. Online* 13:3. 10.1186/1480-9222-13-3 21369534 PMC3047434

[B60] O’Connor E. McCartney K. (2007). Attachment and cognitive skills: An investigation of mediating mechanisms. *J. Appl. Dev. Psychol.* 28 458–476. 10.1016/j.appdev.2007.06.007

[B61] O’Donnell K. A. Gaudreau H. Colalillo S. Steiner M. Atkinson L. Moss E. (2014). The maternal adversity, vulnerability and neurodevelopment project: Theory and methodology. *Can. J. Psychiatry* 59 497–508. 10.1177/070674371405900906 25565695 PMC4168812

[B62] Opie J. E. McIntosh J. E. Esler T. B. Duschinsky R. George C. Schore A. (2020). Early childhood attachment stability and change: A meta-analysis. *Attach. Hum. Dev.* 00 897–930. 10.1080/14616734.2020.1800769 32772822 PMC7612040

[B63] Pruessner J. C. Kirschbaum C. Meinlschmid G. Hellhammer D. H. (2003). Two formulas for computation of the area under the curve represent measures of total hormone concentration versus time-dependent change. *Psychoneuroendocrinology* 28 916–931. 10.1016/S0306-4530(02)00108-7 12892658

[B64] Radolf L. S. (1977). The CES-D Scale A Self report Depression Scale for Research in the General Population. *Appl. Psychol. Meas.* 1 385–401.

[B65] Reilly E. B. Gunnar M. R. (2019). Neglect, HPA axis reactivity, and development. *Int. J. Dev. Neurosci.* 78 100–108. 10.1016/j.ijdevneu.2019.07.010 31374220

[B66] Rispoli K. M. McGoey K. E. Koziol N. A. Schreiber J. B. (2013). The relation of parenting, child temperament, and attachment security in early childhood to social competence at school entry. *J. Sch. Psychol.* 51 643–658. 10.1016/j.jsp.2013.05.007 24060065

[B67] Roque L. Veríssimo M. Oliveira T. F. Oliveira R. F. (2012). Attachment security and HPA axis reactivity to positive and challenging emotional situations in child-mother dyads in naturalistic settings. *Dev. Psychobiol.* 54 401–411. 10.1002/dev.20598 22487942

[B68] Rosenthal R. Rubin D. B. (2003). R equivalent: A simple effect size indicator. *Psychol. Methods* 8, 492–496. 10.1037/1082-989X.8.4.492 14664684

[B69] Rosseel Y. (2012). lavaan: An R package for structural equation modeling. *J. Stat. Softw.* 48:1521. 10.3389/fpsyg.2014.01521 25601849 PMC4283449

[B70] Rubio-Codina M. Grantham-McGregor S. (2020). Predictive validity in middle childhood of short tests of early childhood development used in large scale studies compared to the Bayley-III, the Family Care Indicators, height-for-age, and stunting: A longitudinal study in Bogota, Colombia. *PLoS One* 15:e0231317. 10.1371/journal.pone.0231317 32348359 PMC7190101

[B71] Schore A. N. (2001). Effects of a Secure Attachment Relationship on Right Brain Development, Affect Regulation, and Infant Mental Health. *Infantr Ment. Heal. J. Michigan Assoc* 22 7–66.

[B72] Shakiba N. Raby K. L. (2021). Attachment dimensions and cortisol responses during the strange situation among young children adopted internationally. *Attach. Hum. Dev.* [Epub ahead of print]. 10.1080/14616734.2021.1896445 33719896 PMC8664559

[B73] Siqueira A. C. Betts M. K. Dell’Aglio D. D. (2006). A rede de apoio social e afetivo de adolescentes institucionalizados no sul do Brasil. *Rev. Interam. Psicol.* 40 149–158.

[B74] Tottenham N. Shapiro M. Flannery J. Caldera C. Sullivan R. M. (2019). Parental presence switches avoidance to attraction learning in children. *Nat. Hum. Behav.* 3 1070–1077. 10.1038/s41562-019-0656-9 31332302 PMC7218758

[B75] Van Bakel H. J. A. Riksen-Walraven J. M. (2004). Stress reactivity in 15-month-old infants: Links with infant temperament, cognitive competence, and attachment Security. *Dev. Psychobiol.* 44 157–167. 10.1002/dev.20001 15054884

[B76] Yoshikawa H. Weiland C. Brooks-Gunn J. Burchinal M. R. Espinosa L. M. Gormley W. T. (2013). *Investing in our Future: the Evidence Base On Preschool Education.* New York, NY: Society for Research in Child Development.

[B77] Zeanah C. H. Smyke A. T. Koga S. F. Carlson E. (2005). Attachment in Institutionalized and Community Children in Romania. *Child Dev.* 76 1015–1028. 16149999 10.1111/j.1467-8624.2005.00894.x

[B78] Ziegler G. Dahnke R. Winkler A. D. Gaser C. (2013). Partial least squares correlation of multivariate cognitive abilities and local brain structure in children and adolescents. *Neuroimage* 82 284–294. 10.1016/j.neuroimage.2013.05.088 23727321

